# Heart Failure With Preserved Ejection Fraction: A Case of Cardiac Amyloidosis and the Importance of Echocardiography in the Diagnostic Workup of Infiltrative Disease

**DOI:** 10.7759/cureus.98258

**Published:** 2025-12-01

**Authors:** Prarthna Shah, Kaitlin N Murphy, Szabolcs Simo, Alberth Alvarado, Thomas Vanhecke

**Affiliations:** 1 Medicine, Kansas College of Osteopathic Medicine, Wichita, USA; 2 Internal Medicine, Edward Via College of Osteopathic Medicine, Blacksburg, USA; 3 Internal Medicine, Henry Ford Genesys Hospital, Grand Blanc, USA; 4 Cardiology, Henry Ford Genesys Hospital, Grand Blanc, USA

**Keywords:** amyloidosis, cardiac amyloidosis, echocardiography findings cardiac amyloidosis, heart failure with preserved ejection fraction (hfpef), infiltrative disease

## Abstract

Cardiac amyloidosis (CA) is an uncommon and likely underdiagnosed infiltrative cardiomyopathy that results from extracellular deposition of misfolded proteins. Although considered rare, its true prevalence is believed to be higher than currently recognized due to its nonspecific presentation and diagnostic complexity. Echocardiography remains a cornerstone of the initial evaluation, often providing the earliest clues that suggest underlying CA. Multiple subtypes of CA exist, each posing unique diagnostic challenges and requiring distinct treatment strategies. We describe a previously healthy patient who presented with progressive anasarca and new-onset diabetes mellitus. Initial workup was unrevealing; however, an echocardiogram later demonstrated features concerning for CA, which were promptly identified by an astute clinician. This recognition led to a targeted diagnostic workup and ultimately confirmed the diagnosis. This case report describes a previously healthy patient who developed progressive anasarca and new-onset diabetes. After an echocardiogram was performed for further evaluation, characteristic features of CAs were identified by an astute clinician, leading to appropriate diagnostic testing.

## Introduction

Heart failure with preserved ejection fraction (HFpEF) accounts for approximately 50% of all heart failure cases and presents a growing burden on healthcare systems worldwide [1]. Unlike heart failure with reduced ejection fraction (HFrEF), HFpEF is often more strongly associated with comorbidities such as hypertension, diabetes mellitus, and renal dysfunction. In some cases, however, it may represent a manifestation of underlying infiltrative cardiomyopathy. Cardiac amyloidosis (CA), particularly transthyretin amyloid cardiomyopathy (ATTR-CM) and light chain (AL) amyloidosis, is an underrecognized yet increasingly identified cause of HFpEF [2,3]. The natural course of CA is typically progressive, beginning with asymptomatic diastolic dysfunction, advancing to restrictive cardiomyopathy, and eventually resulting in overt heart failure, arrhythmias, and multi-organ involvement if untreated [4]. Early diagnosis is therefore crucial, as delayed recognition often leads to advanced disease at presentation and limited therapeutic options [5].

Diagnosing CA can be difficult due to its nonspecific clinical features, which may include progressive dyspnea, peripheral edema, and fatigue [6]. Electrocardiogram findings such as low voltage QRS complexes or pseudoinfarction patterns, combined with echocardiographic features such as concentric left ventricular hypertrophy, speckled myocardial texture, and apical sparing on strain imaging, can raise suspicion for infiltrative disease [7,8]. Echocardiography plays a central role in the early identification of CA and can initiate prompt work up with serum and urine free light chain analysis and subsequent confirmatory testing if positive, including cardiac MRI and/or tissue biopsy [9].

This case describes a middle-aged female patient, presenting with acute on chronic anasarca and hypertension, who was ultimately found to have clinical findings highly suspicious for CA. This case underscores the diagnostic utility of echocardiography in evaluating HFpEF and highlights the importance of early recognition of infiltrative cardiomyopathies to initiate appropriate management.

## Case presentation

A 55-year-old female patient with a history of hypertension presented with several months of progressive anasarca and diffuse bilateral edema, initially involving the lower extremities and later extending to the abdomen and forearms. On admission, her blood pressure (BP) was 192/101 mmHg (reference value: <130/80 mmHg). She denied chest pain, dyspnea, or orthopnea, and had no known history of heart failure, thyroid disease, or diabetes. Physical examination revealed significant pitting edema, facial puffiness, and jugular venous distension.

Initial labs were notable for the following (Table 1).

**Table 1:** Significant laboratory results at admission BNP: B-type natriuretic peptide; TSH: Thyroid Stimulating Hormone.

Test/Measurement	Result	Normal range
BNP	850 pg/mL	<100 pg/mL
Serum albumin	2.7 g/dL	3.5-5.0 g/dL
Glucose	384 mg/dL	70-99 mg/dL (fasting)
Hemoglobin A1c	13.20%	<5.7%
TSH	10.84 µIU/mL	0.4-4.0 µIU/mL

**Figure 1:**
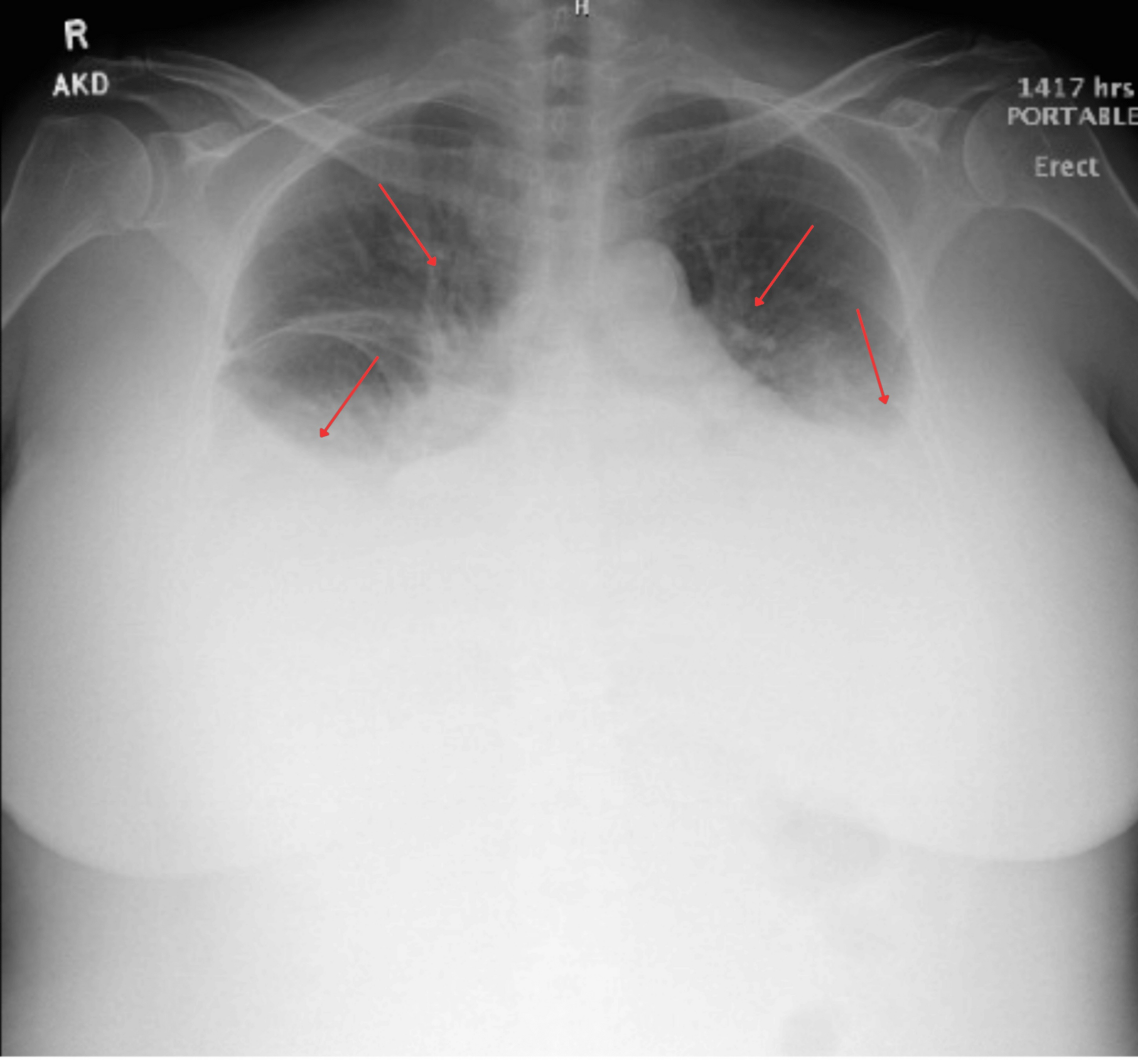
Chest X-ray demonstrating moderate pleural effusions with pulmonary congestion An upright, portable anteroposterior chest radiograph shows bilateral blunting of the costophrenic angles and layering fluid, indicated by red arrows, consistent with moderate pleural effusions. There is also an increased perihilar and interstitial opacity, suggesting pulmonary vascular congestion.

Chest radiograph demonstrated moderate bilateral pleural effusions and mild vascular congestion (Figure 1).

**Figure 2:**
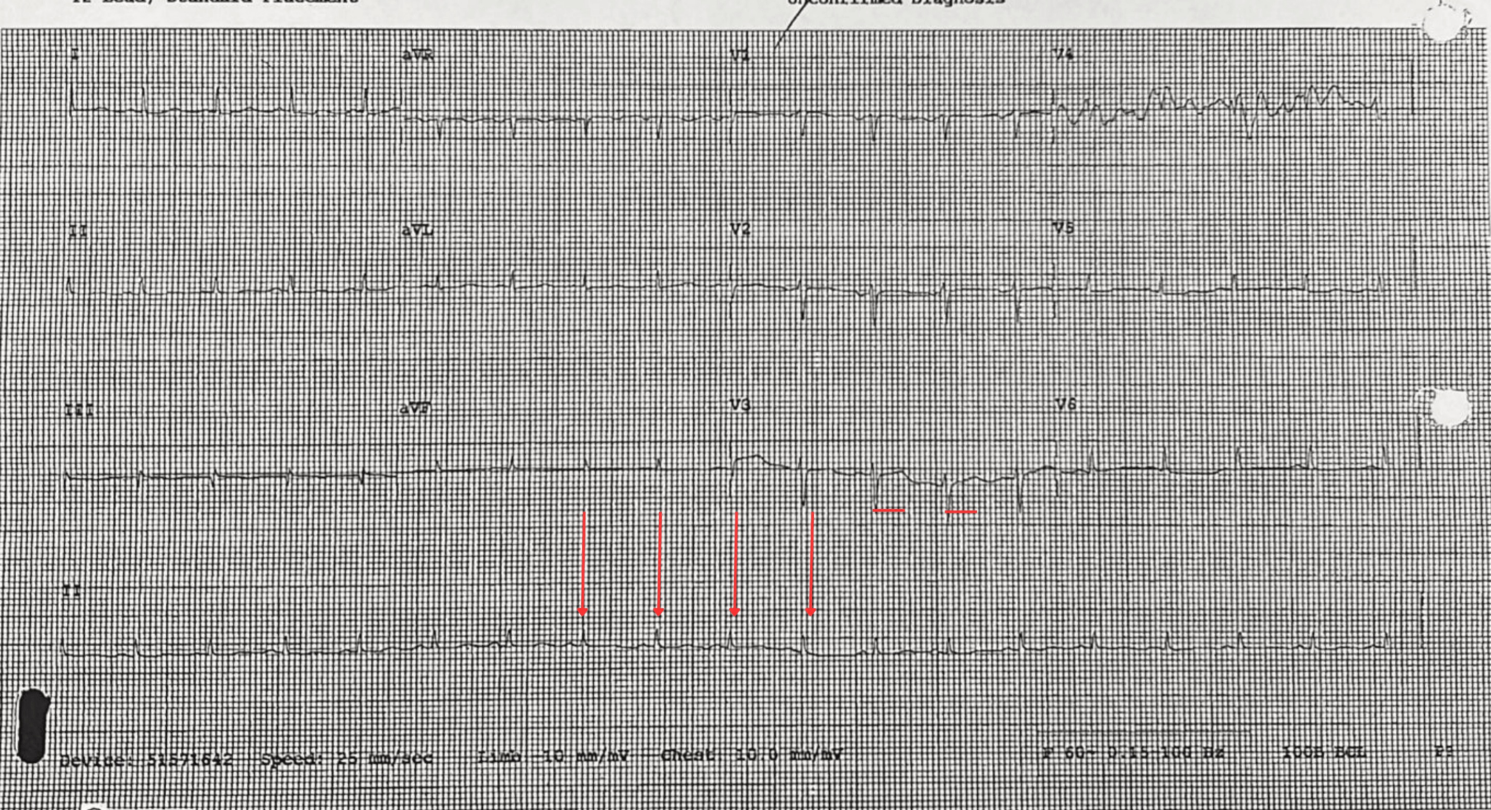
Electrocardiogram showing sinus tachycardia with low-voltage QRS and prolonged QTc The 12-lead ECG demonstrates a sinus tachycardia rhythm with diffusely low-amplitude QRS complexes across the limb leads and a prolonged corrected QT interval (QTc). These findings may raise suspicion for an underlying infiltrative cardiomyopathy.

An electrocardiogram revealed sinus tachycardia with low voltage QRS complexes and a prolonged QTc, which could suggest an infiltrative cardiomyopathy (Figure 2).

**Figure 3:**
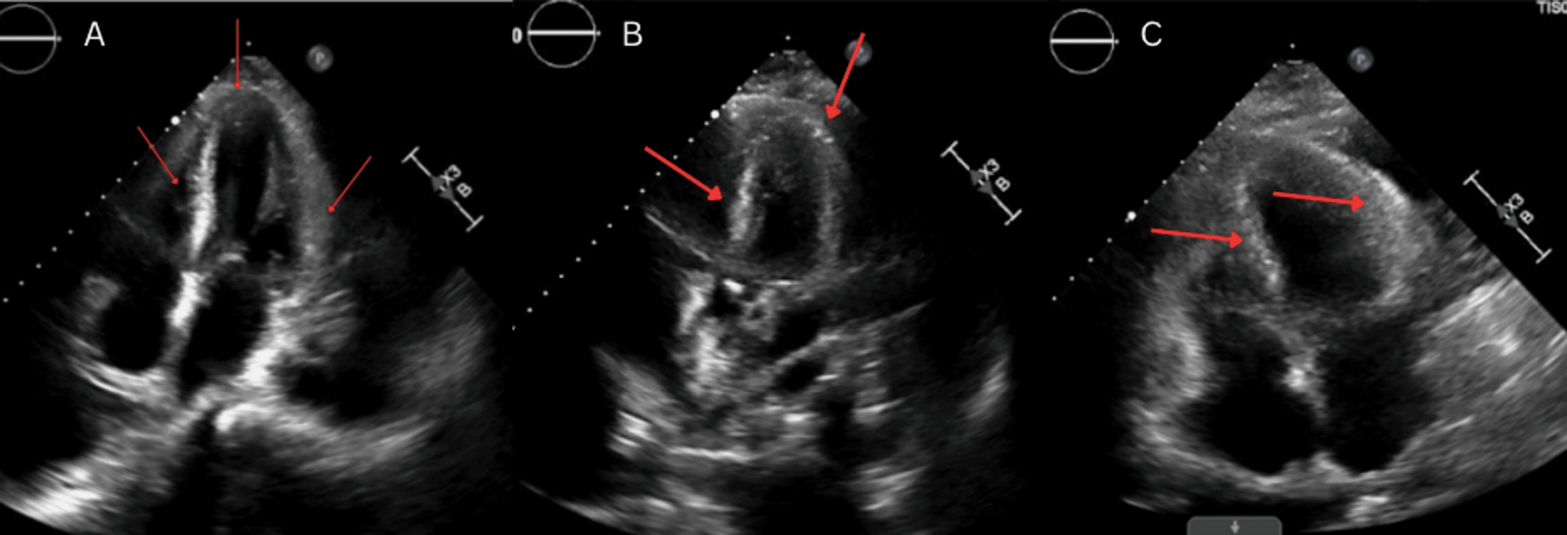
Transthoracic echocardiogram demonstrating findings consistent with cardiac amyloidosis Figure shows apical four-chamber views demonstrating concentric left ventricular hypertrophy and a characteristic “speckled” or granular myocardial appearance (red arrows). Strain imaging revealed an apical sparing pattern with preserved left ventricular ejection fraction (50-55%), classic echocardiographic features concerning for cardiac amyloidosis.

A transthoracic echocardiogram was done, which revealed a preserved left ventricular ejection fraction of 50-55%, concentric left ventricular hypertrophy, and a speckled myocardial appearance with an apical sparing pattern on strain imaging, consistent with features concerning for CA (Figures 3A-3C).

Serum protein electrophoresis showed no monoclonal spike, but free light chain analysis revealed the following, as seen in Table 2.

**Table 2:** Results of the free light chain analysis

Test/Measurement	Result	Normal range
Kappa/Lambda ratio	1.77	0.26 – 1.65
Kappa free light chains	52.7 mg/L	3.3 – 19.4 mg/L
Lambda free light chains	29.8 mg/L	5.7 – 26.3 mg/L

A fat pad biopsy was completed for diagnostic purposes and was negative. Cardiac MRI was ordered for further diagnostic evaluation, but was pending at the time of submission.

The patient was started on oral furosemide, spironolactone, and placed on fluid restriction with close monitoring of fluid balance. Cardiology and hematology were consulted. Her hypertensive urgency was treated with lisinopril, nifedipine, clonidine, and PRN enalaprilat for systolic BP>170 mmHg. Renal ultrasound ruled out renal artery stenosis. Her diabetes was managed with a low-dose insulin sliding scale and long-acting insulin. Endocrinology was consulted for evaluation of subclinical hypothyroidism.

This case highlights a diagnostically challenging presentation of HFpEF, with a strong suspicion of CA, based on characteristic EKG, echocardiographic, and laboratory findings. Ongoing evaluation, including tissue biopsy and cardiac MRI, is critical to confirm the diagnosis and guide treatment.

## Discussion

This case demonstrates an interesting presentation of overt, severe heart failure in an otherwise previously healthy female, emphasizing the importance of maintaining a high index of suspicion for CA in patients presenting with new-onset HFpEF, especially when accompanied by atypical features such as profound anasarca, preserved ejection fraction, low-voltage EKG, and echocardiographic findings of concentric hypertrophy with apical sparing on strain imaging. It is also possible that the patient’s hypertension was not a primary diagnosis but rather a consequence of the infiltrative process itself, as amyloid deposition can increase ventricular stiffness and impair diastolic relaxation, leading to elevated filling pressures and secondary hypertension [10]. These clues should prompt a diagnostic workup for infiltrative cardiomyopathies, which remain frequently underdiagnosed due to their subtle and nonspecific clinical presentation [7]. In older adults, the estimated prevalence of ATTR-CM is actually rising, and can often masquerade as hypertensive heart disease or HFpEF [11,12]. Early recognition is crucial, as timely diagnosis directly impacts therapeutic options, especially with the emergence of disease-modifying treatments such as tafamidis for ATTR and chemotherapy-based regimens for AL amyloidosis [5,8].

The case also reinforces the pivotal role of echocardiography in identifying CA-specific structural changes and also prompting further confirmatory testing, including serum and urine light chain assays, cardiac MRI, and tissue biopsy. The 'apical sparing' pattern on strain imaging has been increasingly recognized as a highly sensitive and specific echocardiographic marker for CA, and may precede other diagnostic markers [13]. Furthermore, because this patient did not have coronary artery disease, the subtle finding of low-voltage QRS complexes on EKG, when seen in conjunction with left ventricular hypertrophy on the echocardiogram, should heighten suspicion for infiltrative disease [14]. This case is also notable for the patient’s simultaneous new diagnoses of diabetes and subclinical hypothyroidism, which while not pathognomonic, can be systemic manifestations of amyloidosis, affecting the pancreas and the thyroid, respectively [15].

From a systems perspective, this case highlights diagnostic blind spots in evaluating HFpEF. Many patients, particularly women and underrepresented minorities, may be misdiagnosed with 'essential hypertension' or 'diabetic cardiomyopathy', when in fact an infiltrative etiology may be present. Because delayed diagnosis remains a major challenge in AL amyloidosis, increased clinical awareness, the use of strain imaging, and attention to red-flag features may help facilitate earlier recognition [16]. Finally, the case also speaks to interdisciplinary collaboration, including prompt involvement of cardiology, hematology, and endocrinology, which allowed for rapid initiation of a targeted diagnostic workup and symptom management, reflecting the need for team-based care in complex presentations such as CA [17]. Given the patient’s subclinical hypothyroidism, it is also worth considering that amyloid infiltration of the thyroid gland may have contributed to endocrine dysfunction, a finding described in both AL and ATTR subtypes [18].

## Conclusions

This case underscores the diagnostic complexity of CA in patients presenting with HFpEF and atypical systemic features, including profound anasarca, new-onset diabetes, and subclinical hypothyroidism. The constellation of low-voltage EKG findings, echocardiographic evidence of concentric hypertrophy with apical sparing, and abnormal light chain ratios highlights the importance of maintaining a high index of suspicion for infiltrative cardiomyopathies. Echocardiography, particularly strain imaging, plays a central role in identifying early disease and guiding subsequent diagnostic pathways, while systemic manifestations may provide additional clues to a multisystem process. As novel therapies such as tafamidis and chemotherapy-based regimens become increasingly available, timely recognition and diagnosis of CA are essential to improving outcomes. Equally important, this case demonstrates the need for multidisciplinary collaboration in complex HFpEF presentations, as coordinated involvement of cardiology, hematology, and endocrinology enables comprehensive evaluation, avoids misdiagnosis, and ensures patients receive appropriate, patient-centered care.
